# Laparoscopic and Endoscopic cooperative surgery as Rescue-treatment for Advanced gastric Cancer in patients Unfit for Surgery (LE-RACUS): protocol for a feasibility study

**DOI:** 10.1186/s40814-024-01584-3

**Published:** 2025-01-03

**Authors:** Henrik Maltzman, Masami Omae, Fredrik Klevebro, Francisco Baldaque-Silva, Ioannis Rouvelas

**Affiliations:** 1 Department of Clinical Science, Intervention and Technology (CLINTEC), Division of Surgery and Oncology, Karolinska Institutet, Hälsovägen 13, 141 57 Huddinge, Stockholm, Sweden; 2 Department of Upper Abdominal Diseases, Karolinska University Hospital, Stockholm, Sweden; 3 Division of Medicine, Department of Upper Gastrointestinal Diseases, Karolinska University Hospital and Karolinska Institute, Stockholm, Sweden; 4 Gastroenterology Department, Advanced Endoscopy Center Carlos Moreira da Silva, Hospital Pedro Hispano, Senhora da Hora, Portugal

**Keywords:** Gastric cancer, Minimally Invasive Surgery, LECS

## Abstract

**Background:**

The standard treatment for advanced gastric cancer without metastasis is gastrectomy in combination with chemotherapy. Some patients cannot tolerate such treatment because of old age or comorbidities. In this study, we want to test the feasibility of Laparoscopic and Endoscopic Cooperative Surgery (LECS) as a less invasive treatment option. In LECS, the tumor margin is marked endoscopically, followed by surgical removal under endoscopic guidance. Currently, LECS is primarily used in Asian countries as a treatment for gastrointestinal stromal cell tumors.

**Methods:**

The study will be conducted as a prospective single-center, feasibility trial. The primary objective will be the safety of LECS, defined as Clavien-Dindo score ≥ III. The secondary objectives will be any complications, postoperative bleeding/perforation, operation time, radicality, mortality, hospital stay, and health-related quality of life.

The inclusion criteria will be patients with gastric cancer cT2-T4aN0M0, Borrman type 1–2 < 5 cm, or type 3 < 2 cm that the tumor board assesses as not fit for gastrectomy. Exclusion criteria will be Borrman type 4 and lesions in the cardia. The patients will be followed up with an outpatient appointment 30 days after the procedure.

**Discussion:**

LECS is a promising treatment option for patients with gastric cancer who cannot tolerate gastrectomy. Compared to gastrectomy, LECS is a less invasive procedure with a documented low complication rate. No previous prospective studies have been conducted to evaluate LECS for advanced gastric cancer.

**Trial registration:**

ClinicalTrials.gov identifier: NCT06105515. Registered 23 October 2023.

https://clinicaltrials.gov/study/NCT06105515?cond=Gastric%20Cancer&term=NCT06105515&aggFilters=status:not%20rec&rank=1

**Supplementary Information:**

The online version contains supplementary material available at 10.1186/s40814-024-01584-3.

## Background

While global incidence rates of gastric cancer are decreasing, the absolute numbers are, in fact, on the rise in many areas of the world because of growing populations [1]. With increasing life expectancy, some high-incidence countries like Japan and Korea are currently observing a higher proportion of elderly patients diagnosed with gastric cancer [2]. Treating this patient group can be challenging due to concomitant disease and frailty.

Patients with early gastric cancer can be treated endoscopically, while those with advanced gastric cancer (AGC) require surgical treatment. In Western countries, most gastric cancers are diagnosed at an advanced stage [3]. The recommended curative intent treatment method for AGC is gastrectomy, employing open or minimally invasive surgery with lymph node dissection in combination with chemotherapy and recently targeted therapies and immunotherapy [4–6]. However, gastrectomy carries a risk for severe complications of up to 20% [7]. Consequently, patients with advanced age or comorbidities may not tolerate such invasive treatment. In these cases, if gastrectomy or palliative oncological treatment cannot be offered, the only remaining option is best supportive care.

In general, survival in gastric cancer is closely linked to the stage of the disease (8). Representatively, the 5-year survival rates for localized, regional, and metastasized disease are 72%, 33%, and 6% respectively [9]. Moreover, the 5-year survival rate in patients with AGC without lymph node or distant metastases who undergo curative intent resection is 69.5–90.9% for T2, 52–77.9% for T3, and 40–71.2% for T4 disease [10]. Patients with AGC who cannot receive definitive surgical or oncological treatment may develop complications such as pain, bleeding from the primary tumor, or gastric outlet obstruction. Such complications can be difficult to manage by endoscopic means and have a significant negative impact on the patient’s quality of life.

Laparoscopic and endoscopic cooperative surgery (LECS) was reported by Hiki et al. in 2008 as a treatment for submucosal tumors [11]. With this method, the endoscopist first performs a mucosal incision around the tumor, followed by laparoscopic removal of the tumor with endoscopic guidance. In Japan, the current indication for LECS is gastrointestinal stromal cell tumors (GIST) with a size of 2–5 cm. LECS has also been described in two case reports as palliative treatment for patients with AGC without being in a state to undergo gastrectomy [12, 13]. To the best of our knowledge, no prospective trial has studied LECS for this indication. Compared to gastrectomy, LECS seems to be a safe and less invasive technique, with fewer severe adverse events [14]. If the tumor could be completely resected with LECS, the risk for bleeding and other tumor-related complications might be reduced, which could probably benefit the patients and improve their quality of life.

The current study aims to investigate if LECS can be performed safely for patients with AGC who are not fit for gastrectomy.

## Methods

Identification and reporting of relevant elements of this protocol are based on the Consolidated Standards of Reporting Trials (CONSORT) extension to pilot and feasibility trials checklist [15] and Template for Intervention Description and Replication (TIDieR) guidelines for intervention descriptions [16] (Supplementary Tables S1 and S2).

This is the first published version of this clinical trial study protocol.

### Study design

The study will be conducted as a single-center, feasibility, prospective non-randomized trial at Karolinska University Hospital, Sweden. Patients will be screened for inclusion through our multidisciplinary tumor board meeting. If they are found to meet the inclusion criteria and after a signed informed consent is obtained, they will be scheduled for an LECS procedure.

### The procedure

An experienced interventional endoscopist and an experienced specialized upper gastrointestinal surgeon from the team will perform the intervention under general anesthesia. First, the tumor is endoscopically visualized, and the margin is marked with a diathermia probe. A circumferential mucosal incision is then made around the marked area. Concomitantly, laparoscopy is performed, and the stomach is exposed. To avoid spillage of gastric juices and possible contamination into the abdominal cavity, sutures are placed around the tumor to circumferentially pull up the area into a crown shape. Under laparoscopic visualization, the endoscopist intentionally perforates the gastric wall. The tumor is then resected laparoscopically along the endoscopic incision line. Lastly, the tumor is removed using an Endocatch retrieval pouch, and the gastric defect is sutured. No lymphadenectomy is performed. If, for any reason, the procedure cannot be completed laparoscopically, the surgery will be aborted, and no resection will be performed.

### Postoperative care

Postoperatively, the patient will be hospitalized and observed for a few days in our inpatient ward. Thirty days after surgery, an outpatient clinic meeting will be scheduled. At this follow-up, a physical exam will be performed, as well as a full review of the medical charts. The patients will also be asked to fill out two health-related quality-of-life questionnaires, QLQ-C30 and OG25.

### Scientific objectives

The objective of the current study is to investigate the safety and feasibility of LECS as an alternative treatment for AGC in patients unfit for gastrectomy with lymphadenectomy.

The primary outcome of the study will be the safety of LECS, defined as Clavien-Dindo complication grade ≥ III [17].

The secondary outcome measures will be the following:

- Any complication (Clavien-Dindo II–IV)
- Postoperative bleeding requiring blood transfusion
- Leakage/postoperative abscess requiring drainage
- Operation time
- Local radicality
- Health-related quality of life (QLQ-C30/OG25 scores)
- 30-day mortality,
- In-hospital mortality
- Hospital-stay

### Study population

Patients with AGC referred to our institution will be eligible for inclusion in the study if they are unfit for standard treatment with gastrectomy and lymphadenectomy.

### Inclusion criteria

The inclusion criteria are as follows:

- cT2-T4aN0M0 gastric carcinoma according to the TNM classification, the eighth edition established by the Union for International Cancer Control [18].
- Borrmann type 1–2 < 5 cm or Borrmann type 3 < 2 cm.
- Patient assessment by the multidisciplinary tumor board as not fit for gastrectomy
- Signed informed consent.

### Exclusion criteria

The exclusion criteria are as follows:

- Borrmann type 4.
- Location in the cardia.

### Assessment of safety

Complications will be continuously monitored for each procedure according to the clinic’s routines. An interim analysis will be performed for each of the 3 cases completed. If any death is observed directly attributed to the procedure, the study will be prematurely discontinued. Moreover, the study will also be discontinued if severe complications (Clavien-Dindo complication grade ≥ III) are observed in > 20% of cases, which is the expected rate for gastrectomy [7].

### Follow-up

All the patients will be followed according to the clinic’s routines, namely with a first postoperative control in the outpatient clinic 30 days after the procedure and then every 3 months for 1 year. For patients who die before the end of follow-up, the date of death will be registered (Table 1).

**Table 1:** Eligible patients’ flowchart

Patients eligible for inclusion	Preoperative visit	LECS^a^-procedure	Postop visit (30 days)	Every 3rd month up to a year
Information and consent form	x	x		
Physical examination	x	x	x	x
HRQL^b^-questionnaires	x	x	x	x
Review of medical charts		x	x	x

^a^*LECS* Laparoscopic Laparoscopic and Endoscopic Cooperative Surgery

^b^*HRQL* health-related quality of life

### Sample size

We aim to include 15 patients in the study.

### Sample size justification

This is a feasibility study, so power calculation is deemed unnecessary.

### Management of data

Information and data from patients included in the study will be handled only by researchers working under confidentiality according to the Health and Healthcare Act. The data will be collected in a database and pseudonymized with study-specific patient codes. All study documentation will be stored in a locked, protected location intended for handling confidential information at Karolinska University Hospital in Stockholm. Only personnel delegated for the study, as well as the monitor, will have, under confidentiality protection, access to patient records and source data. The code key in these cases will be handled by the appointed research coordinator at the Medical Unit of Upper Abdomen, Karolinska University Hospital in Stockholm. The data material will be destroyed 10 years after publication.

### Ethics

The study is conducted in compliance with the protocol and in accordance with the ethical principles of the Second Declaration of Helsinki and with Good Clinical Practice rules. The EU General Data Protection Regulation (GDPR) will be respected. The rights, safety, and well-being of the trial subjects are the most important considerations and should prevail over the interests of science and society. Study personnel conducting this trial are qualified by education, training, and experience to perform their respective tasks.

Ethical approval has been granted from the Swedish Ethical Review Authority in Stockholm, Sweden, with reference numbers 2023–04699-01 and 2023–06744-02.

### Dissemination of knowledge and publication strategy

The study has been registered with ClinicalTrials.gov, NCT06105515. Positive as well as negative or inconclusive results will be submitted for publication in international peer-reviewed journals and oral presentations at relevant national and international congresses.

## Discussion

Compared with gastrectomy, LECS is a minimally invasive procedure for local tumor control in the stomach. The method has been previously widely studied in Asian countries for the management of GISTs and other submucosal tumors and has been found to have favorable outcomes [19–23]. In this study, we want to test the feasibility of LECS for AGC in patients with advanced age or comorbidities that make them unsuitable for the recommended standard surgical treatment. The optimal management of elderly patients with gastric cancer is currently being scrutinized in various studies. In a recent article by Sekiguchi et al., it is noted that the 90-day mortality after gastrectomy is gradually increasing from 0,4% in the age group ≤ 74 years to 5.5% in the age group ≥ 90 years. Furthermore, 20% or more of elderly patients die within 5 years after gastrectomy, often due to other diseases (24). In another Japanese study from 2017, postoperative complications were an independent risk factor for survival in patients ≥ 75 years but not in younger patients [25]. The authors’ conclusion was to consider limited surgery instead of radical surgery in this patient group.

Additionally, given the poor prognosis, if no treatment can be offered, a palliative care approach that aims to improve quality of life and decrease suffering by anticipating, preventing, and treating pain and other symptoms and problems is particularly important for patients with gastric cancer [26]. In this study, we want to test if LECS is a viable option that could minimize patient risk while upholding a good quality of life. If this first trial has a positive outcome, it could lead to future, larger studies on the subject. Our next step would be to conduct a phase 2 trial comparing the best supportive care to local resection with the primary outcome of health-related quality of life and the secondary outcome of long-term survival.

A possible problem with the current study will primarily be patient inclusion. Sweden is a low-incidence country for gastric cancer, and almost half of the newly diagnosed cases have a generalized disease [27]. Furthermore, the strict inclusion criteria make patient inclusion even more difficult. We hope to include 15 patients over a 2-year period; however, the timeframe may need to be extended if cases are limited.

Another treatment option for patients unfit for gastrectomy is palliative chemotherapy under the condition that the patient can tolerate it. Whether LECS will be an alternative treatment for this group of patients is something to be investigated in the future. Finally, because the study will be conducted on a frail, high-risk patient group, it is important to monitor the safety of the procedure continuously, and the trial will be immediately terminated if any death is observed directly attributed to the procedure.

## Supplementary Information

Supplementary Material 1: Supplementary Table S1. The TIDieR (Template for Intervention Description and Replication) Checklist*.

Supplementary Material 2: Supplementary Table S2. CONSORT 2010 checklist of information to include when reporting a pilot or feasibility trial*

## Data Availability

Once the study has been completed and the findings have been reported, pilot data will be available from the corresponding author upon reasonable request.

## Abbreviations

AGC: Advanced gastric cancer
LECS: Laparoscopic and Endoscopic Cooperative surgery
GIST: GastroIntestinal stromal cells tumors
